# Histological reappraisal of IgA nephropathy: the role of glomerular pattern of injury and mesangial complement deposition

**DOI:** 10.1186/s12882-024-03577-z

**Published:** 2024-04-24

**Authors:** Bogdan Obrișcă, Valentin Mocanu, Roxana Jurubiță, Alexandra Vrabie, Andreea Berechet, Ștefan Lujinschi, Bogdan Sorohan, Andreea Andronesi, Camelia Achim, Gabriela Lupușoru, Georgia Micu, Nicu Caceaune, Mihaela Gherghiceanu, Gener Ismail

**Affiliations:** 1 “Carol Davila” University of Medicine and Pharmacy, Bucharest, Romania; 2 Department of Nephrology, Fundeni Clinical Institute, Bucharest, Romania; 3 Department of Internal Medicine, Fundeni Clinical Institute, Bucharest, Romania; 4 “Victor Babes” National Institute of Pathology, Bucharest, Romania

**Keywords:** IgA nephropathy, Renal outcome, Histology, Glomerular pattern, Complement

## Abstract

**Background:**

There is a clear need to refine the histological assessment in IgA Nephropathy (IgAN). We sought to investigate the clinical significance of the light microscopy (LM) pattern of glomerular injury and of the intensity of mesangial C3 staining in IgAN.

**Methods:**

We conducted a retrospective, observational study that included all patients with biopsy-proven primary IgAN that had at least 12 months of follow-up. The LM pattern of glomerular injury was reevaluated based on a modified HAAS classification. Mesangial C3 deposition by immunofluorescence (IF) staining was scored semi-quantitatively. The study primary composite endpoint was defined as doubling of serum creatinine or ESRD (dialysis, renal transplant or eGFR < 15 ml/min). The secondary study endpoint was eGFR decline per year.

**Results:**

This cohort included 214 patients with IgAN (mean age, 41.4 ± 12.6 years), with a mean eGFR and median 24-h proteinuria of 55.2 ± 31.5 ml/min/1.73m^2^ and 1.5 g/day (IQR:0.8–3.25), respectively. The most frequent LM pattern was the mesangioproliferative (37.4%), followed by the sclerotic (22.5%) and proliferative/necrotizing patterns (21.4%). Regarding the IF findings, mild-moderate and intense mesangial C3 staining was present in 30.6% and 61.1% of patients, respectively. Those with sclerosing and crescentic patterns had the worst renal survival (5-year renal survival of 48.8% and 42.9%) and the highest rate of eGFR change/year (-2.32 ml/min/y and − 2.16 ml/min/y, respectively) compared to those with other glomerular patterns of injury. In addition, those with intense C3 staining reached the composite endpoint more frequently compared to those without intense C3 staining (35.5% vs. 21.4%, *p* = 0.04). After multivariate adjustment, patients with crescentic and sclerosing patterns had a 3.6-fold and 2.1-fold higher risk for the composite endpoint compared to those with mesangioproliferative pattern, while an intense mesangial C3 deposition being also associated with a worse renal outcome (HR, 3.33; 95%CI, 1.21–9.2).

**Conclusions:**

We have shown that the LM pattern of glomerular injury and the intensity of mesangial C3 deposition might stratify more accurately the renal outcome in patients with IgAN.

**Supplementary Information:**

The online version contains supplementary material available at 10.1186/s12882-024-03577-z.

## Background

Immunoglobulin A nephropathy (IgAN) is the most common primary glomerular disease worldwide [1]. Although it was generally accepted that up to 50% of patients with IgAN progress to end-stage renal disease (ESRD) within 20 years from diagnosis, recent data has challenged our initial perspective that some patients may have a “benign” clinical course. Thus, a recent study showed that almost all patients are at risk of progression to ESRD within their expected lifetime unless an estimated glomerular filtration rate (eGFR) loss below 1 ml/min/year is achieved [2]. This data must be interpreted in the context of the recent results from randomized controlled trials evaluating either optimized supportive care or non-specific immunosuppression: the rate of renal function decline was − 1.6 ml/min/y (optimized supportive care arm of the STOP-IgAN trial), -3.5 ml/min/y (dapagliflozin arm of the DAPA-CKD trial), -1.4 ml/min/y (immunosuppression arm of the STOP-IgAN trial) and − 2.5 ml/min/y (methylprednisolone arm of the TESTING trial) [3–5]. Thus, with current therapies the rate of eGFR decline remains unacceptably high [6].

The current prognostication of primary IgAN relies on the International IgAN Prediction Tool that incorporates the Oxford Classification and clinical variables measured at the time of biopsy [7]. However, relying on the Oxford Classification to characterize the renal risk may oversimplify the histological picture in IgAN, given that IgAN is characterized by a diversity of glomerular and tubulointerstitial lesions that may influence renal survival [1]. In addition, among the Oxford Classification variables only the extent of tubular atrophy/interstitial fibrosis (IFTA) has been consistently validated across studies as an independent predictor of renal outcome, while for the glomerular lesions there is a significant inconsistency, a poor reproducibility among pathologists and the association with renal survival remains uncertain [1, 8]. Nonetheless, the severity of IFTA is invariably associated with renal outcome irrespective of the underlying etiology of a glomerular disease [9, 10]. Moreover, the current Oxford Classification cannot be used as a treatment stratification algorithm [11].

Given that the treatment landscape of IgAN has recently encountered a tremendous progress with several new agents that target various pathogenic pathways showing a benefit in randomized controlled trials, it became obvious that there is a need to better individualize prognostication and guide therapy relying on factors additional to proteinuria and renal function [12–16]. Thus, patients with incipient lesions might benefit from the targeted-release formulation (TRF) of budesonide, while patients with proliferative lesions might need initially more aggressive regimens (e.g.: systemic steroids either in monotherapy or associated with other immunosuppressants, anti-complement therapies in those with signs of intense complement activation by alternate or lectin pathways). This approach may be regarded as reminiscent of that for patients with lupus nephritis (LN). Accordingly, patients with LN show a weak correlation between clinical features and histological findings and the tissue-based information is essential to guide the immunosuppressive therapy [17]. While the current treatment stratification in IgAN relies solely on clinical variables (persistent proteinuria and eGFR level), the possibility of incorporation the tissue-based information into treatment-selection algorithms, while appealing, needs to be tested in prospective, randomized clinical trials.

Nonetheless, while combinations of Oxford Classification variables might reflect different underlying light microscopy (LM) patterns of glomerular injury, the evaluation of all possible combinations is impractical both in clinical practice and in a research setting. As such, whether the LM pattern of glomerular injury might better reflect the clinical characteristics and renal outcome in IgAN remains uncertain. In addition, several other immunofluorescence, light or electron microscopy features, not included in the Oxford Classification, have shown to be associated with renal outcome [1]. Thus, there is a clear need to refine the histological assessment in IgAN. Accordingly, the current KDIGO guidelines state that refining the risk stratification in IgAN remains an area of research of high priority [11].

We sought to investigate the clinical significance of the LM patterns of glomerular injury and of the intensity of mesangial C3 staining in IgAN and their impact on renal outcome.

## Methods

### Study design and population

We conducted a retrospective, observational study that included all patients with biopsy-proven primary IgAN between 1999 and 2022 that had at least 12 months of follow-up. Those with ages under 18 years, whose renal biopsy specimen contained less than 8 scorable glomeruli, with potential secondary causes of IgAN, with insufficient clinical data or a shorter than 12 months of follow-up were excluded from the analysis, leaving a final cohort of 214 patients. The diagnosis of IgAN was based in all patients on immunofluorescence (dominant or codominant IgA in the mesangium), light and electron microscopy examination (paramesangial electron-dense deposits) [18]. All patients underwent a systematic screening for disorders reported to be associated with IgAN [19].

The study was conducted with the provisions of the Declaration of Helsinki and the protocol was approved by the local ethics committee (The Ethics Council of Fundeni Clinical Institute, IRB: 23,250, date of approval: May 9th 2019). The need for informed consent was waived due to exclusive use of deidentified information and the retrospective nature of the study. Informed consent has been waived by the Ethics Council of Fundeni Clinical Institute (IRB: 23,250, date of approval: May 9th 2019).

### Clinical and histological parameters

The clinical variables obtained by reviewing the patient’s medical records at the time of kidney biopsy were age, sex, mean arterial pressure (MAP), treatment with renin-angiotensin-aldosterone system (RAAS) inhibitors and immunosuppressive (IS) therapy. Given the retrospective nature of the study, the treatment of patients with IgAN was conducted at the discretion of the attending physician without any intervention. Laboratory data included evaluation of renal function [serum creatinine and estimated glomerular filtration rate (eGFR) according to the 2009 CKD-EPI equation] [20], serum albumin, serum total IgA and C3, hematuria and 24-h proteinuria. Increased serum IgA and decreased C3 were defined as levels over 400 mg/dL and below 90 mg/dL, respectively. The IgA/C3 ratio was derived from individual serum IgA and C3 values. In addition to absolute values, hematuria was also scored semi-quantitatively as: absent (< 25 cells/µL), mild (25–50 cells/µL), moderate (50–100 cells/µL) and severe (> 100 cells/µL).

The LM pattern of glomerular injury was reevaluated based on a modified Haas classification as following [21]:

- Normal glomeruli - the glomeruli show no more than a minimal mesangial hypercellularity (less than 50% of the glomeruli), without segment sclerosis or proliferative lesions.
- Mesangioproliferative glomerulonephritis– more than 50% of the examined glomeruli show mesangial hypercellularity, without segmental sclerosis or proliferative lesions.
- Proliferative and/or necrotizing glomerulonephritis– glomeruli with mesangial hypercellularity and proliferative lesions (endocapillary and/or extracapillary hypercellularity). Patients included in this category had endocapillary hypercellularity of any severity, but if extracapillary hypercellularity was present it was required to be encountered in less than 50% of the examined glomeruli.
- Crescentic glomerulonephritis– more than 50% of the glomeruli show cellular or fibrocellular crescents.
- Focal or diffuse sclerosing glomerulonephritis– glomeruli with sclerotic lesions (either segmental sclerosis or global sclerosis), without proliferative lesions.

Mesangial C3 deposition by immunofluorescence (IF) staining was scored semi-quantitatively as: absent, mild (1+), moderate (2+) and intense (3+). The intensity score was assigned based on the objective used to detect the signal on a Leica widefield fluorescence microscope. The signal visible with the 10x objective was categorized as intense (3+), moderate if visible with the 20x objective (2+), and mild if visible only with the 40x objective (1+). In addition, we quantified the percentage of glomeruli that were globally sclerosed and the IgA staining by IF (either alone or co-deposition with IgM/IgG).

In addition, all renal biopsy specimens were independently reviewed and scored according to the 2016 revised Oxford Classification by two experienced pathologists [22]. If any disagreement of the histologic assessment between the pathologists was identified, the slides were reevaluated by both and discussed until a final agreement on LM pattern, Oxford Classification and IF staining scores was made.

### Study endpoints

The study primary composite endpoint was defined as doubling of serum creatinine or ESRD (dialysis, renal transplant or eGFR < 15 ml/min), whichever came first. The secondary study endpoint was eGFR decline per year.

### Statistical analysis

Continuous variables were expressed as either mean (± standard deviation or 95% confidence interval) or median (interquartile range: 25th-75th percentiles), according to their distribution, and categorical variables as percentages. Differences between groups were assessed in case of continuous variables by Student t test, Mann–Whitney test, one-way ANOVA or Kruskal–Wallis test, according to the distribution of dependent variables and the level of independent variable, and in case of categorical variables by Pearson χ2 test or Fisher’s exact test.

The probability of event-free survival was assessed by Kaplan-Meier method and the log-rank test was used for comparisons. Univariate and multivariate (Cox proportional hazard ratio) analyses were performed to identify independent predictors of the composite endpoint. The results of Cox analyses are expressed as a hazard ratio (HR) and 95% confidence interval (95% CI).

In all analyses, *p* values are two-tailed and all *p* values less than 0.05 were considered statistically significant.

Statistical analyses were performed using the SPSS program (SPSS version 20, Chicago, IL), and GraphPad Prism version 9.3.1 (1992–2021 GraphPad Software, LLC).

## Results

### Study population

The characteristics of the study population are described in Table 1. This cohort included 214 patients with IgAN (66.4% males) with a mean age at the time of kidney biopsy of 41.4 ± 12.6 years. The mean serum creatinine and corresponding eGFR were 2.02 ± 1.57 mg/dl and 55.2 ± 31.5 ml/min/1.73m^2^, respectively, with 59.4% of patients having an eGFR below 60 ml/min/1.73m^2^. The median 24-h proteinuria was 1.5 g/day (IQR:0.8–3.25), with 22.9% of patients having nephrotic-range proteinuria (> 3.5 g/day). An increased serum level of total IgA and a decreased serum C3 level were encountered in 27.9% and 12.9% of cases, respectively, with the mean IgA/C3 ratio being 3.18 ± 1.62.

**Table 1:** Baseline characteristics of the study cohort

Variable	Value
**Number of patients**	214
**Age (years)**	41.4 ± 12.6
**Sex (male, %)**	66.4%
**Mean arterial pressure (mmHg)**	99 ± 15
**Serum creatinine (mg/dL)**	2.02 ± 1.57
**eGFR (ml/min/1.73m**^**2**^)	55.2 ± 31.5
**CKD stage (%)**	
• G1	15.8%
• G2	24.8%
• G3	34.1%
• G4	19.2%
• G5	6.1%
**Albumin (g/dL)**	4.07 ± 0.63
**Serum IgA (mg/dL)**	351 ± 153
**Increased serum IgA (% pf pts.)**	27.9%
**Serum C3 (mg/dL)**	116 ± 24
**Decreased serum C3 (% of pts.)**	12.9%
**IgA/C3 ratio**	3.18 ± 1.62
**Hematuria (cells/µL)**	35 (14–84)
**Hematuria (% of pts)**	
• Absent	37.2%
• Mild	28%
• Moderate	15.5%
• Severe	19.3%
**24-h proteinuria (g/24 h)**	1.5 (0.8–3.25)
**24-h proteinuria (% of pts)**	
• Proteinuria < 0.75 g/24 h (%)	22.9%
• Proteinuria 0.75–3.5 g/24 h (%)	54.2%
• Proteinuria > 3.5 g/24 h (%)	22.9%
**Treatment**	
• RAAS inhibitors (%)	88%
• Corticosteroids monotherapy (%)	46.2%
• Corticosteroids ± other immunosuppressive agent (%)	25.5%
**Outcome**	
• Doubling of serum creatinine (%)	18.2%
• ESRD (%)	24.8%
• Combined endpoint (%)	29%
**Follow-up (months)**	49.1 (17.3–86.2)

Abbreviations: eGFR, estimated glomerular filtration rate; CKD, chronic kidney disease; RAAS, renin-angiotensin-aldosterone system; ESRD, end-stage renal disease

In terms of histological findings, the most frequent LM pattern of glomerular injury identified was the mesangioproliferative pattern (37.4%), followed by the sclerotic pattern (22.5%) and proliferative/necrotizing glomerulonephritis (21.4%) (Table 2). Approximately 15% of the study cohort had normal glomeruli when examined by light microscopy, while only 3.3% showed crescentic glomerulonephritis. Regarding the IF findings, mild-moderate and intense mesangial C3 staining was present in 30.6% and 61.1% of patients, respectively. Isolated IgA staining was present in 21.8% of cases, while the majority had either IgG or IgM co-deposition (39.1% each).

**Table 2:** Histologic characteristics of the study cohort

Variable	Value
**Global glomerulosclerosis (% of glomeruli)**	17.6% (0–35)
**LM pattern of glomerular injury (% of pts)**	
• Normal glomeruli	15.4%
• Mesangioproliferative glomerulonephritis	37.4%
• Proliferative/necrotizing glomerulonephritis	21.5%
• Crescentic glomerulonephritis	3.3%
• Sclerosing glomerulonephritis	22.4%
**Oxford Classification (% of patients)**	
**Mesangial hypercellularity (M1)**	74.8%
**Endocapillary hypercellularity (E1)**	24.3%
**Segmental sclerosis (S1)**	58.4%
**IFTA (T)**	
• T0 (≤ 25%)	56.5%
• T1 (26–50%)	27.6%
• T2 (> 50%)	15.9%
**Crescents (C)**	
• C0	79.9%
• C1	14%
• C2	6.1%
**C3 staining on IF**	
• Absent	8.3%
• Mild-Moderate	30.6%
• Intense	61.1%
**IgA staining on IF**	
• Alone	21.8%
• IgA + IgG co-deposition	39.1%
• IgA + IgM co-deposition	39.1%

Abbreviations: pts, patients; LM, light microscopy, M, mesangial hypercellularity; E, endocapillary hypercellularity; S, segmental sclerosis; IFTA, tubular atrophy and interstitial fibrosis; C, crescents; IF, immunofluorescence

When evaluating the patients by the Oxford Classification, 74.8% of patients had mesangial hypercellularity, 24.3% had endocapillary hypercellularity, 58.4% had segmental sclerosis and 43.5% had at least 25% of the cortical area with IFTA. Crescents were present in at least one glomerulus in 20.1% of patients. The median percentage of glomeruli with global sclerosis was 17.6% (IQR: 0–35).

The majority of patients received a RAAS inhibitor (88%), 46.2% had received steroid monotherapy and approximately 25% received steroids plus other IS agents during the observation period. Regarding the combined IS regimens, the majority of patients received steroids plus cyclophosphamide (43.4%), followed by steroids plus mycophenolate mofetil (33.9%), steroids plus azathioprine (20.8%) and steroids plus rituximab (1.9%).

### Relation of the LM pattern of injury and the intensity of mesangial C3 staining with clinical parameters

The LM pattern of glomerular injury correlated with the severity of IgAN at the moment of presentation (Table 3). The renal function was worse in those with proliferative/necrotizing, crescentic and sclerosing patterns of glomerular injury. Significant differences between LM patterns were also noted for hematuria and proteinuria, patients with proliferative lesions having a higher 24-h proteinuria, while those with crescentic glomerulonephritis having the highest level of proteinuria and hematuria, by comparison to the rest of subgroups. Moreover, the majority of patients with crescentic IgAN (85.7%) had nephrotic syndrome at the moment of kidney biopsy and were significantly younger compared to the other subgroups. In addition, two patients were considered to have an IgAN associated with minimal-change disease after electron microscopy examination. These patients had nephrotic syndrome at presentation, had no mesangial hypercellularity or any proliferative lesions, had mild mesangial C3 staining and were classified as having normal glomeruli by LM. Moreover, they showed a rapid response to steroid therapy with a normal renal function and complete remission of proteinuria on long-term follow-up.

**Table 3:** Univariate analysis according to light microscopy pattern of glomerular injury

Variable	Normal glomeruli	Mesangioproliferative	Proliferative /Necrotizing	Crescentic	Sclerosing	*p*-value
**Number of patients**	33	80	46	7	48	
**Clinical parameters**						
**Age (years)**	41 ± 14.4	42.9 ± 13.6	40.4 ± 11	33.3 ± 15.1	41.1 ± 10.4	0.35
**Sex (male, %)**	54.5%	75%	63%	28.6%	68.8%	0.04
**Mean arterial pressure (mmHg)**	91 ± 14	100 ± 15	102 ± 13	103 ± 10	101 ± 16	0.02
**Serum creatinine (mg/dL)**	1.56 ± 1.87	1.72 ± 0.93	2.15 ± 1.26	2.06 ± 1.24	2.7 ± 2.2	< 0.001
**eGFR (ml/min/1.73m**^**2**^)	74 ± 29	59 ± 30	49 ± 31	54 ± 33	40 ± 25	< 0.001
**CKD stage (%)**						
• G1	36.4%	17.5%	8.7%	14.3%	6.2%	0.001
• G2	33.3%	30%	26.1%	28.6%	8.3%	
• G3	27.3%	32.5%	30.4%	28.6%	45.8%	
• G4	3%	18.8%	21.7%	14.3%	29.2%	
• G5	0%	1.2%	13%	14.3%	10.4%	
**Albumin (g/dL)**	4.2 ± 0.6	4.1 ± 0.5	3.9 ± 0.7	2.8 ± 1.02	4.1 ± 0.4	0.001
**Serum IgA (mg/dL)**	371 ± 182	379 ± 183	329 ± 86	246 ± 84	322 ± 107	0.37
**Increased serum IgA (% pf pts.)**	25%	40.4%	16.7%	0%	20.7%	0.09
**Serum C3 (mg/dL)**	120.3 ± 30.6	115.4 ± 24.2	114.7 ± 21.6	132.3 ± 26.6	113 ± 22.5	0.39
**Decreased serum C3 (% of pts.)**	13.8%	12.7%	13.8%	0%	13.9%	0.91
**IgA/C3 ratio**	3.2 ± 1.6	3.5 ± 2.1	3.02 ± 0.8	1.9 ± 0.4	2.9 ± 0.9	0.15
**Hematuria (cells/µL)**	30 (15–49)	36 (17–97)	35 (15–83)	903 (117–1925)	22 (7–44)	0.002
**Hematuria (% of pts)**						
• Absent	30.3%	35.9%	31.8%	0%	54.3%	0.003
• Mild	45.5%	23.1%	29.5%	16.7%	23.9%	
• Moderate	12.1%	27.9%	20.5%	0%	10.9%	
• Severe	12.1%	23.1%	18.2%	83.3%	10.9%	
**24-h proteinuria (g/24 h)**	0.8 (0.2–1.6)	1.3 (0.7–2.8)	2.2 (1.2–4.5)	8.7 (4.9–11)	1.7 (1.03–3.2)	< 0.001
**24-h proteinuria (% of pts)**						
• Proteinuria < 0.75 g/24 h(%)	48.3%	24.4%	9.8%	0%	19.6%	< 0.001
• Proteinuria 0.75–3.5 g/24 h (%)	41.4%	59%	53.7%	14.3%	60.9%	
• Proteinuria > 3.5 g/24 h (%)	10.3%	16.7%	36.6%	85.7%	19.6%	
***Treatment***						
• RAAS blockade(%)	87.9%	89.7%	86%	71.4%	89.4%	0.68
• Corticosteroids monotherapy (%)	33.3%	50%	41.9%	0%	59.6%	< 0.001
• Corticosteroids ± other IS agents (%)	12.1%	16.7%	46.5%	100%	19.1%	
***Histological parameters***						
Global glomerulosclerosis (% pf glomeruli)	0% (0–19)	5.8% (0-28.5)	20% (0–33)	9.3% (0-16.6)	53.8% (33.3–66.6)	< 0.001
Intense C3 staining on IF (% of patients)	29.6%	67.6%	64.9%	57.1%	68.3%	0.009
IgA staining on IF (% of patients)						
• Alone	40.7%	25%	14.7%	0%	12.5%	0.11
• IgA + IgG co-deposition	33.3%	42.6%	35.3%	40%	40%	
• IgA + IgM co-deposition	26%	32.4%	50%	60%	47.5%	
Arteriolar hyalinosis (% of patients)	60.6%	59.2%	58.1%	71.4%	93.5%	0.001
Arteriosclerosis (% of patients)	33.3%	21.1%	23.3%	28.6%	52.2%	0.006
***Outcome***						
• Doubling of serum creatinine (%)	0%	12.5%	21.7%	57.1%	31.2%	< 0.001
• ESRD (%)	0%	12.5%	34.8%	57.1%	47.9%	< 0.001
• Combined endpoint (%)	0%	18.8%	34.8%	71.4%	54.2%	< 0.001
eGFR decline*						
• eGFR change/y (ml/min/1.73m^2^/y)	+ 1.2 (-0.79 to 9.03)	-0.29 (-3.02 to 1.87)	-0.45 (-2.83 to 3.97)	-2.32 (-10.3 to 3.71)	-2.16 (-6.01 to -0.07)	< 0.001
• Percentage eGFR change (%)	+ 7.1% (3.45 to 30.7)	-4% (-12.9 to 7.31)	-4.27% (-12.3 to 27.6)	-43.3% (-87.4 to 49.8)	-26.4% (-36.3 to 14.2)	< 0.001
• eGFR decline > 5 ml/min/y (% of pts.)	3%	16%	16.3%	42.9%	37.8%	0.001

* Analysis after exclusion of patients with rapid progression to ESRD (≤ 12 months)

Abbreviations: pts., patients; eGFR, estimated glomerular filtration rate; CKD, chronic kidney disease; RAAS, renin-angiotensin-aldosterone system; ESRD, end-stage renal disease; IF, immunofluorescence

**Fig. 1:**
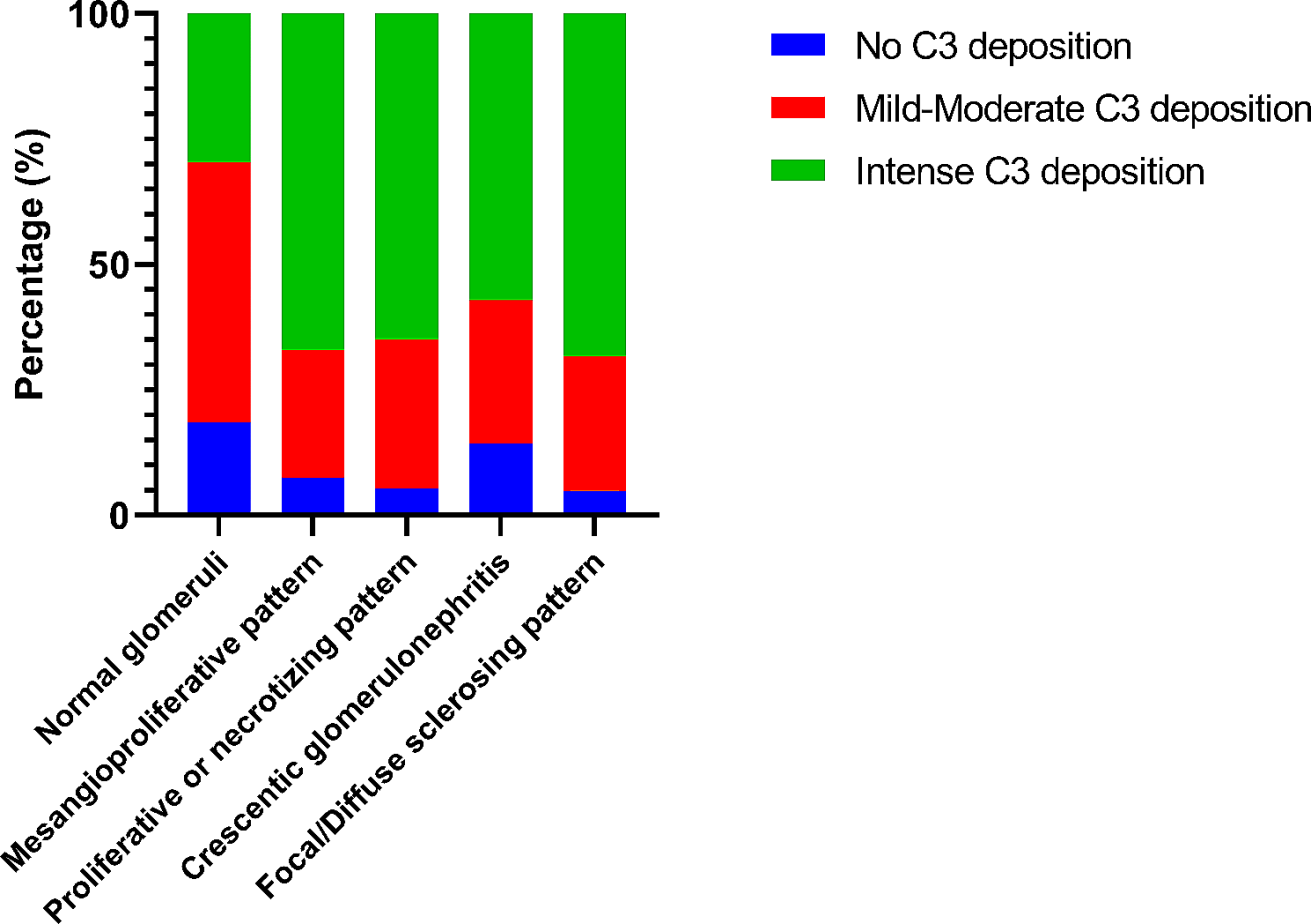
Relation of mesangial C3 deposition with light microscopy patterns of glomerular injury

In terms of histological findings, the mesangial C3 deposition was associated with the development of both proliferative and chronic patterns of glomerular injury. By comparison to those with normal glomeruli by LM, patients with other LM patterns had more frequently intense mesangial C3 staining (67.6% for mesangioproliferative pattern, 64.9% for proliferative/necrotizing pattern, 57.1% for crescentic pattern and 68.3% for sclerotic pattern vs. 29.6% for normal glomeruli pattern) (Fig. 1). In addition, the prevalence of isolated IgA deposition was highest in those with normal glomeruli, while IgG or IgM co-deposition was seen in all patients with crescentic IgAN. Vascular lesions were more prevalent in those with crescentic or sclerosing patterns of injury.

When evaluating the impact of mesangial C3 deposition, we identified that the only significant clinical parameter associated with the intensity of mesangial C3 staining was the level of 24-h proteinuria (Supplemental Table 1). Patients with intense mesangial C3 staining had a higher proteinuria compared to those without intense C3 staining [1.7 g/day (IQR:0.5–3.1) vs. 1.2 (IQR: 0.8–3.6), *p* = 0.05]. In addition, despite not reaching statistical significance, these patients had a tendency for a worse renal function and a higher MAP at the moment of kidney biopsy. There was no relation between the serum level of C3, total IgA or IgA/C3 ratio and the intensity of mesangial C3 staining.

In terms of histological findings, the intensity of C3 deposition was mainly associated with chronic lesions (Supplemental Table 1). Those with intense C3 staining had more frequently mesangial hypercellularity (81.8% vs. 64.3%, *p* = 0.008), segmental sclerosis (64.5% vs. 51.4%, *p* = 0.08) and a greater prevalence of global glomerulosclerosis (median percentage of glomeruli with global sclerosis, 20.7% vs. 16.6%, *p* = 0.11), compared to those without intense C3 staining. In addition, the extent of IFTA was associated with mesangial C3 deposition, approximately 50% of those with intense C3 staining having a T1-2 score compared to 28.5% in those without intense staining (*p* = 0.006). More patients with strong mesangial C3 deposition had coexisting IgA-IgM co-deposition compared to those with weaker mesangial C3 deposition (44.9% vs. 29.9%, *p* = 0.03). Nonetheless, mesangial C3 deposition was not associated with proliferative lesions (endocapillary hypercellularity or crescents).

### The impact of histological parameters on renal outcome

During a median follow-up period of 49.1 months (IQR:17.3–86.1), a total of 29% of the study cohort reached one of the events of the primary composite endpoint, with 24.8% eventually progressing to ESRD. The median time for progression to ESRD was 24.4 months (IQR: 10.7–69.7), with 19 patients (8.9%) progressing to ESRD in less than 12 months following the kidney biopsy. Patients that progressed had worse renal function, higher proteinuria and higher MAP at baseline compared to those that did not reach the composite endpoint (Supplemental Table 1). In addition, serum C3 levels were lower in those that reached the composite endpoint.

**Fig. 2:**
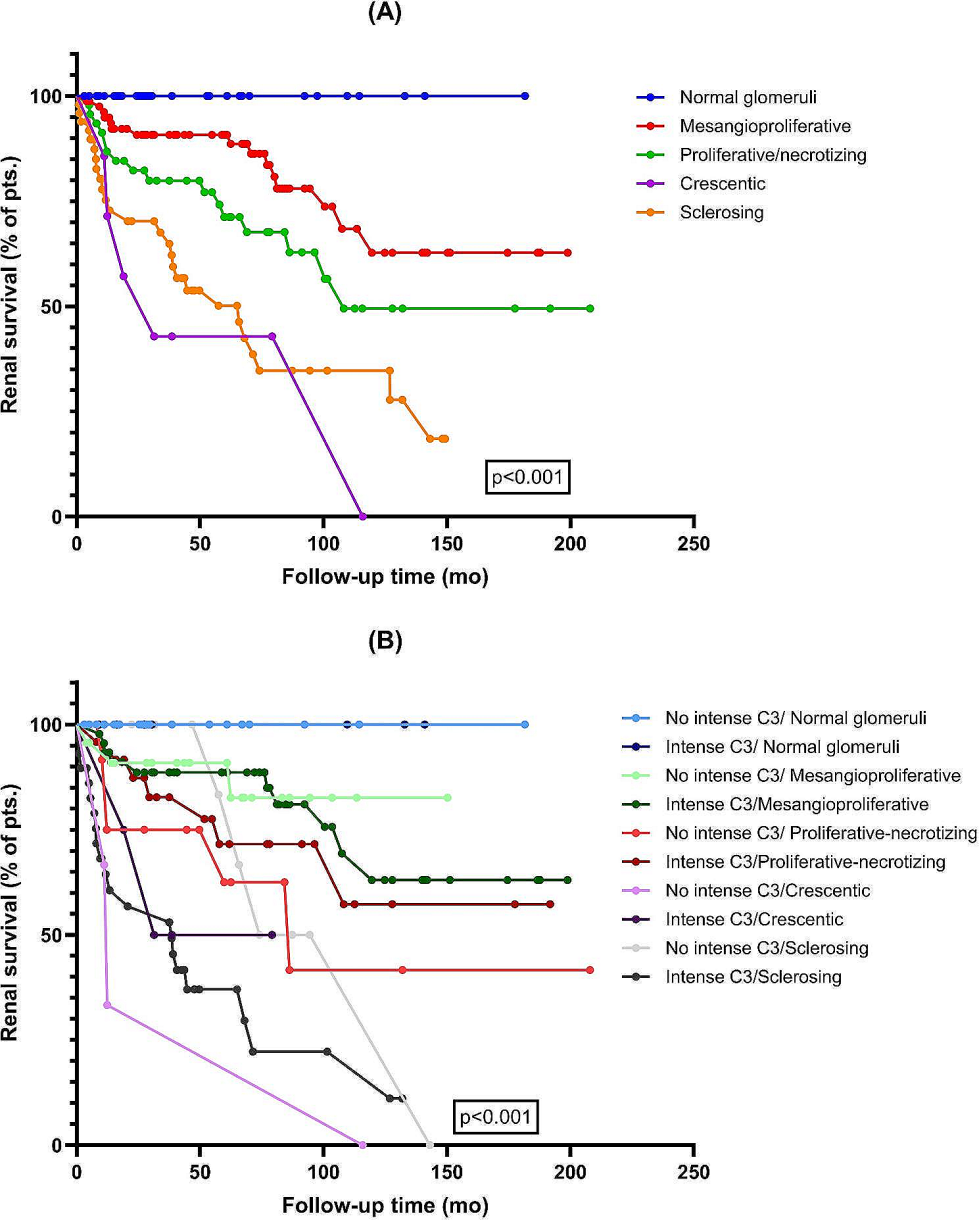
Renal survival in relation to light microscopy pattern alone and stratified by the intensity of mesangial C3 deposition

**Fig. 3:**
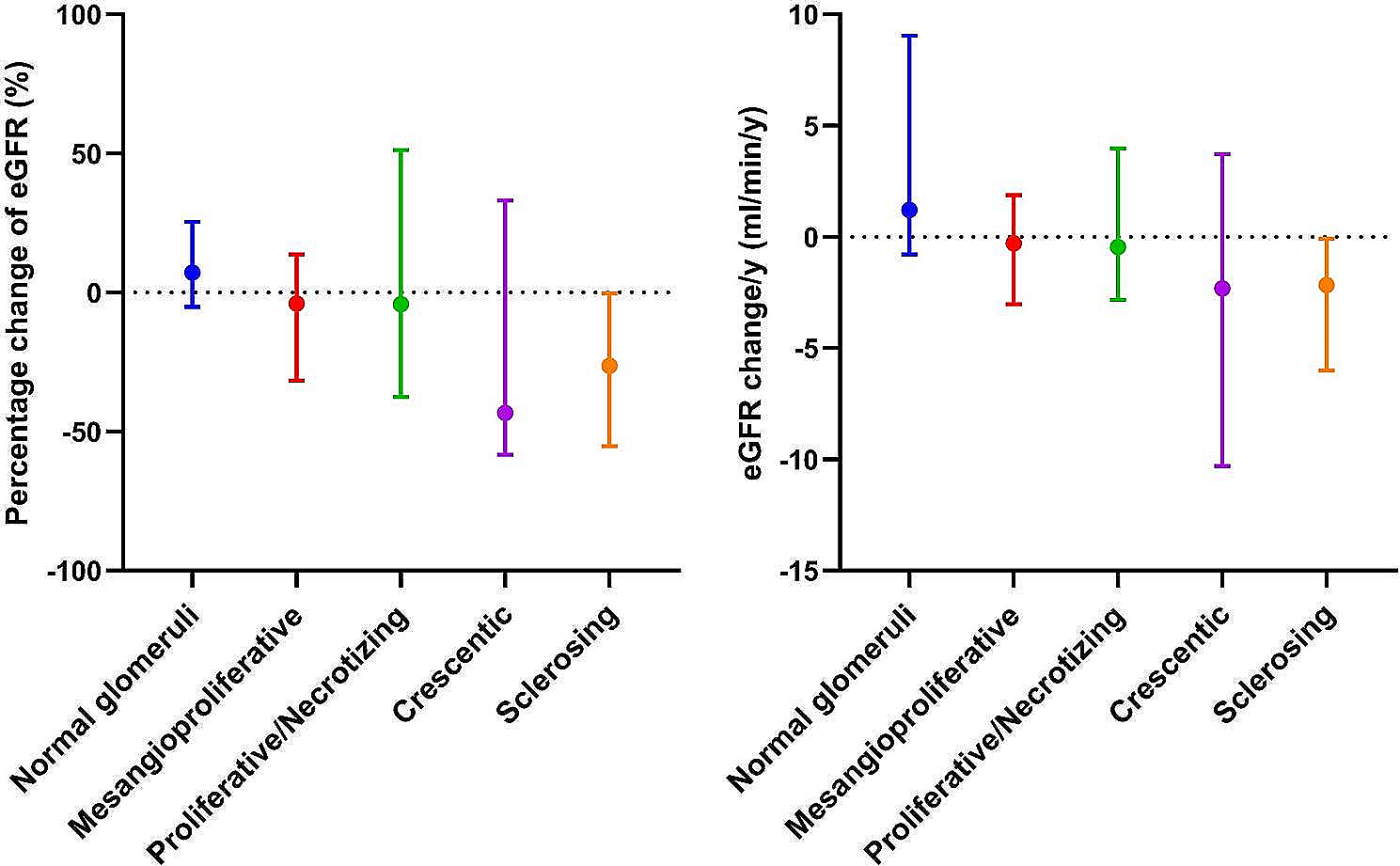
Renal function change according to light microscopy patterns of glomerular injury

In terms of the LM pattern, those with sclerosing and crescentic pattern had the worst renal survival. The 5-year renal survival according to the LM pattern was: 100% for normal glomeruli, 90.7% for mesangioproliferative pattern, 71.2% for proliferative/necrotizing pattern, 42.9.% for crescentic pattern and 48.8% for sclerotic pattern (Supplemental Table 2, Fig. 2). After stratifying the renal survival analysis according to both LM pattern/Oxford Classification variables and mesangial C3 deposition, we noted that patients with intense C3 staining had lower renal survival, with the most significant differences being noticed in those with T2 score (5-year renal survival, 80% vs. 23.6%, *p* = 0.04) and sclerotic LM pattern (5-y renal survival, 83.3% vs. 34.5%, *p* = 0.007) (Supplemental Table 2, Fig. 2). After restricting the analysis to those that did not progress to ESRD within 12 months from biopsy, patients with crescentic and sclerosing patterns showed a higher rate of eGFR change/year (-2.32 ml/min/y and − 2.16 ml/min/y, respectively) and a higher percentage change in eGFR (-43.3% and − 26.4%, respectively) compared to those with other glomerular patterns of injury (Table 3; Fig. 3).

The intensity of mesangial C3 staining was also associated with renal outcome, those with intense C3 staining reaching the composite endpoint or ESRD more frequently compared to those without intense C3 staining (35.5% vs. 21.4%, *p* = 0.04 and 30.9% vs. 15.7%, *p* = 0.02, respectively). After restricting the analysis to those that did not progress to ESRD within 12 months from biopsy, patients with intense C3 staining showed a higher rate of eGFR change/year and a higher percentage change in eGFR compared to those without intense C3 staining [-1.4 ml/min/year vs. +3.03 ml/min/y, *p* = 0.02 and − 8.4% vs. +7.61%, *p* = 0.01, respectively]. Despite not reaching statistical significance, more patients with intense C3 staining showed an eGFR decline of more than 5 ml/min/y (20.4% vs. 12.9%, *p* = 0.22) (Supplemental Table 2).

**Fig. 4:**
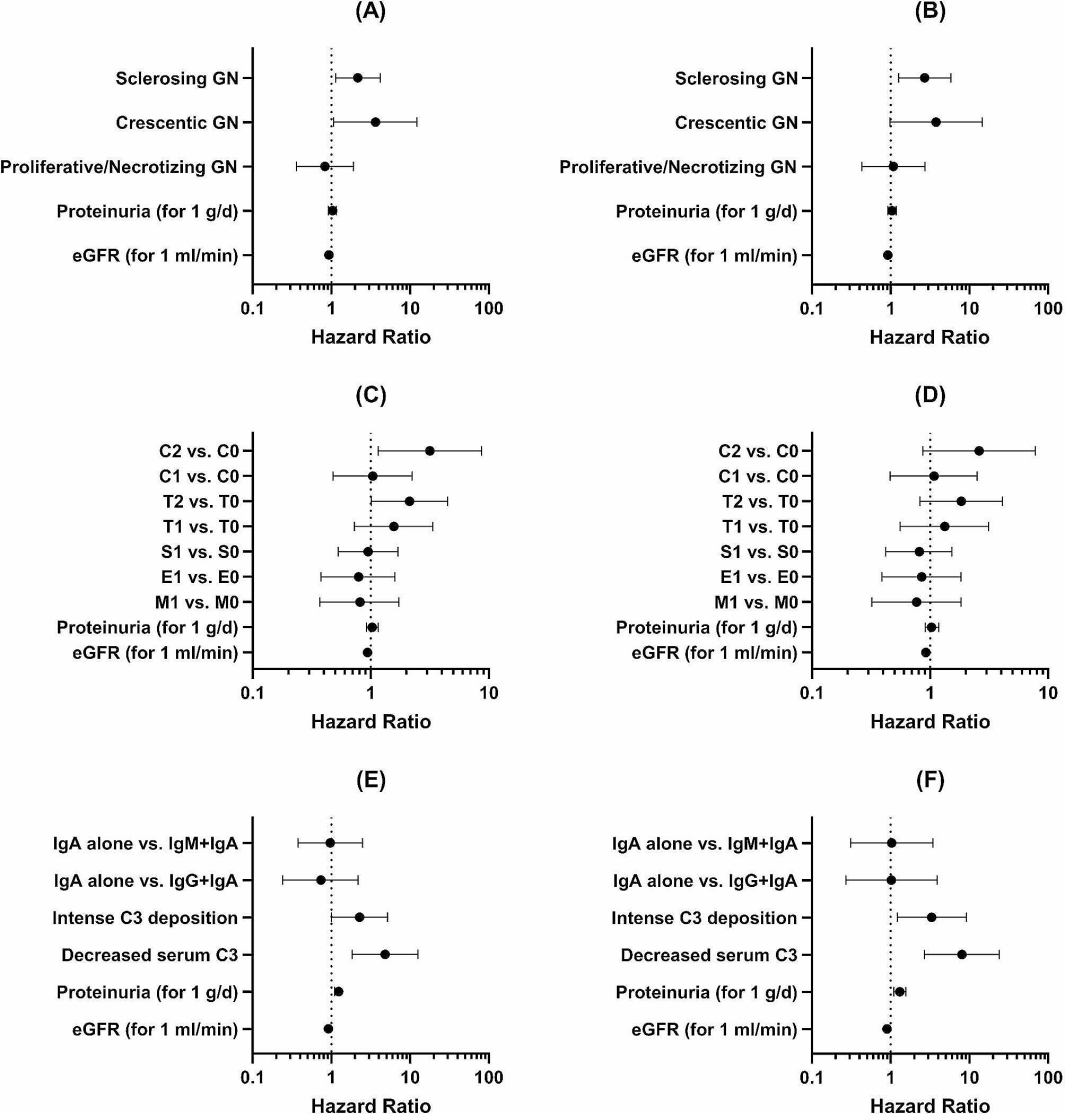
Predictive factors of renal outcome. **A, C, E**) Composite endpoint. **B, D, F**) ESRD

The results of the univariate and multivariate Cox proportional hazard regression analysis to identify the independent predictors of renal outcome are depicted in Table 4. For multivariate analysis several models were evaluated (Fig. 4). The renal function at baseline was consistently identified as an independent predictor of renal outcome in all models employed. In addition, in the LM model 1 that accounted for the LM pattern of injury, by comparison to the mesangioproliferative pattern, patients with crescentic and sclerosing patterns had a 3.6-fold and 2.1-fold higher risk for the composite endpoint. In the LM model 2 that incorporated Oxford Classification variables, the T2 score (HR, 2.13; 95%CI, 1.01–4.47) and C2 score (HR, 3.17; 95%CI, 1.16–8.7) were identified as independent predictors of worse renal outcome. However, if we restricted the analysis to ESRD as the outcome event, the Oxford Classification variables could not identify the patients with a higher risk for renal disease progression. In the IF model, the intensity of complement activation predicted renal outcome, with an intense mesangial C3 deposition and a decreased serum C3 level being associated with a higher risk for ESRD [(HR, 3.33; 95%CI, 1.21–9.2) and (HR, 8.05; 95CI%, 2.7-24.04), respectively].

**Table 4:** Univariate and multivariate cox proportional hazards regression analysis regarding predictive factors of renal outcome

Variable	ESRD		Composite endpoint	
	Hazard Ratio (95%CI)	*p*-value	Hazard Ratio (95%CI)	*p*-value
***Univariate analysis***				
Age (for 1 y)	1.003 (0.98–1.02)	0.76	1.01 (0.99–1.03)	0.33
Sex (male vs. female)	1.02 (0.56–1.84)	0.94	1.07 (0.62–1.86)	0.79
MAP (for 1 mmHg)	1.02 (1.009–1.04)	0.003	1.02 (1.01–1.04)	0.001
eGFR (for 1 ml/min/1.73m2)	0.91 (0.89–0.93)	< 0.001	0.93 (0.91–0.95)	< 0.001
Proteinuria (for 1 g/day)	1.16 (1.06–1.26)	0.001	1.14 (1.06–1.24)	0.001
Oxford Classification				
• M1 vs. M0	1.48 (0.69–3.14)	0.31	1.36 (0.69–2.68)	0.37
• E1 vs. E0	1.34 (0.75–2.38)	0.32	1.15 (0.66-2)	0.6
• S1 vs. S0	1.18 (0.67–2.06)	0.55	1.31 (0.78–2.2)	0.31
• T1 vs. T0	2.82 (1.42–5.62)	0.003	2.97 (1.59–5.55)	0.001
• T2 vs. T0	8.38 (4.27–16.4)	< 0.001	7.85 (4.18–14.7)	< 0.001
• C1 vs. C0	2.71 (1.44–5.11)	0.002	2.4 (1.31–4.37)	0.004
• C2 vs. C0	5.68 (2.58–12.5)	< 0.001	5.18 (2.47–10.8)	< 0.001
Decreased serum C3 (vs. no decreased)	2.85 (1.32–6.14)	0.007	2.13 (1.02–4.47)	0.04
Intense C3 deposition (vs. no intense)	1.72 (0.87–3.41)	0.11	1.43 (0.78–2.59)	0.24
IgA co-deposition (vs. IgA alone)	-	-	-	-
• IgG + IgA	1.01 (0.36–2.83)	0.97	0.68 (0.28–1.62)	0.38
• IgM + IgA	2.01 (0.76–5.33)	0.15	1.49 (0.67–3.31)	3.31
LM pattern (vs. mesangioproliferative)	-	-	-	-
• Proliferative/necrotizing	2.91 (1.32–6.42)	0.008	1.96 (0.97–3.97)	0.06
• Crescentic	6.38 (1.99–20.4)	0.002	5.31 (1.92–14.6)	0.001
• Sclerosing	5.74 (2.72–12.1)	< 0.001	4.4 (2.32–8.34)	< 0.001
***Multivariate analysis***				
***A) Light microscopy model 1***				
eGFR (for 1 ml/min/1.73m2)	0.92 (0.89–0.94)	< 0.001	0.93 (0.92–0.95)	< 0.001
Proteinuria (for 1 g/day)	1.04 (0.92–1.17)	0.53	1.04 (0.92–1.16)	0.48
LM pattern (vs. mesangioproliferative)	-	-	-	-
• Proliferative/necrotizing	1.08 (0.43–2.73)	0.85	0.83 (0.36–1.91)	0.65
• Crescentic	3.77 (0.98–14.5)	0.05	3.64 (1.07–12.3)	0.03
• Sclerosing	2.7 (1.26–5.79)	0.01	2.17 (1.13–4.19)	0.02
***B) Light microscopy model 2***				
eGFR (for 1 ml/min/1.73m2)	0.92 (0.9–0.95)	< 0.001	0.94 (0.92–0.96)	< 0.001
Proteinuria (for 1 g/day)	1.03 (0.91–1.18)	0.61	1.03 (0.92–1.16)	0.56
Oxford Classification	-	-	-	-
• M1 vs. M0	0.77 (0.32–1.83)	0.56	0.81 (0.37–1.73)	0.58
• E1 vs. E0	0.85 (0.39–1.82)	0.68	0.79 (0.38–1.6)	0.51
• S1 vs. S0	0.81 (0.42–1.53)	0.51	0.95 (0.53–1.7)	0.88
• T1 vs. T0	1.33 (0.56–3.13)	0.51	1.57 (0.73–3.35)	0.24
• T2 vs. T0	1.84 (0.82–4.11)	0.13	2.13 (1.01–4.47)	0.04
• C1 vs. C0	1.08 (0.46–2.51)	0.85	1.04 (0.48–2.24)	0.91
• C2 vs. C0	2.61 (0.87–7.82)	0.08	3.17 (1.16–8.7)	0.02
***C) Immunofluorescence model***				
eGFR (for 1 ml/min/1.73m2)	0.9 (0.86–0.94)	< 0.001	0.92 (0.89–0.95)	< 0.001
Proteinuria (for 1 g/day)	1.31 (1.1–1.55)	0.002	1.26 (1.1–1.24)	0.001
Decreased serum C3 (vs. no decreased)	8.05 (2.7-24.04)	< 0.001	4.83 (1.85–12.6)	0.001
Intense C3 deposition (vs. no intense)	3.33 (1.21–9.2)	0.02	2.28 (1.008–5.18)	0.04
IgA co-deposition (vs. IgA alone)	-	-	-	-
• IgG + IgA	1.02 (0.27–3.91)	0.96	0.74 (0.24–2.19)	0.58
• IgM + IgA	1.03 (0.31–3.44)	0.95	0.97 (0.38–2.49)	0.96

Abbreviations: eGFR, estimated glomerular filtration rate; CKD, chronic kidney disease; RAAS, renin-angiotensin-aldosterone system; ESRD, end-stage renal disease; LM, light microscopy, M, mesangial hypercellularity; E, endocapillary hypercellularity; S, segmental sclerosis; IFTA, tubular atrophy and interstitial fibrosis; C, crescents; IF, immunofluorescence; MAP, mean arterial pressure; y, years

## Discussion

In this study, we have shown that the LM pattern of glomerular injury and the intensity of mesangial C3 deposition might reflect better the clinical characteristics of patients with IgAN and stratify more accurately the renal outcome compared to the Oxford Classification. As such, refining the histologic assessment may aid in the renal risk stratification along with the currently accepted risk factors in IgAN.

While the Oxford Classification of IgAN has been originally developed in 2009, little progress has been made in the past decade regarding the histologic stratification of these patients [23]. This should be interpreted in the context of the significant progress made in the understanding of IgAN pathogenesis that led to the development of several new agents targeting various pathogenic pathways: the dual endothelin-angiotensin receptor blocker sparsentan, TRF-budesonide, inhibitors of B-cell activating factors or anti-complement therapies [12, 13, 15, 24, 25]. Accordingly, the “*one-size-fits-all*” treatment selection based on eGFR and proteinuria does not fulfill the needs of patients with IgAN as neither renal function nor proteinuria cannot accurately differentiate between histologic activity or chronicity, or between the need of more intensified optimized supportive care (with the addition of SGLT2 inhibitors or sparsentan) or of more intensified IS treatment.

While the most consistently validated among the Oxford Classification variables is the severity of IFTA (T score), it does not reflect the severity of coexisting glomerular lesions. In order to overcome the limitations of the Oxford Classification, we attempted to evaluate the impact of LM pattern of glomerular injury and to define the role of mesangial C3 deposition in IgAN. This approach may be viewed as similar to LN, where IS treatment is guided by the ISN/RPS class and prognostication takes into account individual lesions encompassed in the NIH activity and chronicity index [17, 26]. In this regard, the mesangioproliferative pattern of IgAN, which resembles class II LN, had the best renal outcome among the proliferative patterns with a 5-year renal survival over 90% and a mean eGFR decline of -0.29 ml/min/y. Given that in this setting the glomerular lesions are not severe and systemic IS therapy may not be necessarily needed, these patients may actually benefit from targeted, local therapies, such as TRF-budesonide [27]. While the rationale of this approach is sustained from a pathogenic standpoint, Coppo R suggesting that targeting the mucosal immune system dysregulation may be most suitable for earlier phases of IgAN before severe kidney damage occurs, it needs to be tested in dedicated trials [27]. Nonetheless, TRF-budesonide has shown clinically relevant reductions in proteinuria and eGFR decline leading to its approval as the first disease-modifying agent in IgAN [16, 28]. However, the current data does not offer the possibility for a further pathologic stratification of the indication of TRF-budesonide and for the selection of which patients would benefit the most from this agent from a histologic standpoint.

The proliferative/necrotizing pattern of glomerular injury had an intermediate outcome in our study, with a 5-year renal survival of 71% and a mean eGFR decline of -0.45 ml/min/y, while almost 90% of patients received systemic steroids (either alone or in association with other IS agents). However, whether these patients may benefit from systemic immunosuppression (other than steroids) remains currently a matter of ongoing debate without a definitive proof from randomized controlled trials. As an example, the trials with mycophenolate mofetil were successful mainly in Asian populations with IgAN and the results have not been replicated to the same extent in other populations [29, 30]. Nonetheless, in the study by Hou et al., that enrolled IgAN patients with proliferative lesions (approximately 40% with endocapillary hypercellularity, approximately 85% with crescents in less than 50% of the glomeruli and approximately 60% with fibrinoid necrosis), addition of mycophenolate mofetil to a lower steroid dose led to similar complete remission rates compared to full-dose prednisone [30]. However, the use of other systemic immunosuppressive agents remains controversial and is restricted by the KDIGO guidelines to certain subpopulations [1, 11]. Despite that a previous analysis of the VALIGA cohort showed that steroids either in monotherapy or in association with other immunosuppressants where more likely to be used in patients with an eGFR below 50 ml/min and in those with proliferative lesions (such as endocapillary hypercellularity), this approach needs to be tested in an adequate prospective, randomized clinical trial setting [31].

A distinct category of IgAN patients is represented by those with crescentic glomerulonephritis, characterized by severe nephrotic syndrome and the worst renal outcome with a 5-year renal survival of 43% and a mean eGFR decline of -2.32 ml/min/y. Current guidelines suggest that these patients should be treated similar to ANCA-associated vasculitis (AAV) [11]. However, the rapidly progressive glomerulonephritis in IgAN seems to be significantly different from AAV, given that the majority of patients present with severe nephrotic syndrome that is poorly responsive to aggressive immunosuppression consisting of systemic steroids and cyclophosphamide. Lastly, the sclerosing pattern should be taken into account in patients with IgAN, similar to class VI LN, highlighting the need for more intensified nephroprotective measures (e.g., addition of SGLT2 inhibitors or the dual endothelin-angiotensin receptor blocker). In terms of chronicity, the Oxford Classification relies on T and S scores, with the presence of segmental sclerosis being inconsistently associated with renal outcome and having a moderate reproducibility among pathologists [8, 22]. The inability of S score to reliably stratify renal outcome might be related to the absence of an adequate threshold to reflect the severity of glomerular involvement with segmental and/or global sclerosis. Thus, defining a sclerosing pattern in IgAN identifies a subcategory of patients with distinct clinical features and different renal outcome. Nonetheless, while our study does not intend to provide treatment suggestions based on the LM patterns of glomerular injury, there is a need for a personalized approach to IgAN taking into consideration pathology and pathogenesis [27].

In addition to LM pattern, we have further evaluated whether the intensity of complement activation as defined by IF staining influences renal survival. The lectin and alternative pathways of the complement system have emerged as key mediators of kidney injury in IgAN that paved the way for the development of complement pathway inhibitors [32]. Accordingly, several studies have outlined that serum level of complement components or glomerular deposition of their fragments may be associated with a worse renal outcome, although with conflicting results [33]. In our study, the intensity of complement activation was mainly associated with chronic lesions, while those with intense C3 staining had a worse renal survival and a higher eGFR decline. As such, patients with intense C3 staining and either a sclerosing pattern or a T2 score had the worst 5-year renal survival (34.5% and 23.6%, respectively). The reason for the association of the intensity of C3 staining mainly with chronicity might be related to the late diagnosis of IgAN in our cohort (with a mean age at the moment of kidney biopsy of over 40 years, a high prevalence of renal events and a short time for progression to ESRD). Other studies have confirmed our findings showing an association of intense mesangial C3 deposition with chronic lesions (S1 or T1-2) [33]. The association of mesangial hypercellularity with mesangial C3 deposition should be interpreted in the context that complement activation represents a key driver of autoimmune-mediated kidney injury. This is supported in our study by the observation that those with predominantly normal glomeruli had weaker C3 staining identified by IF. Nonetheless, whether an earlier diagnosis would have identified a greater prevalence of active lesions and a stronger association with complement activation remains speculative. However, the prevalence of renal events in our cohort is similar to that observed in the major randomized controlled trials, suggesting that we included patients with IgAN with a similar high risk of progression. In the TESTING trial, after a mean follow-up of 4.2 years, 117 out of the 503 (23.2%) patients that underwent randomization progressed to ESRD [4].

Our study has several limitations that need to be acknowledged. First, this is a single center, retrospective study, and our findings need to be replicated in different populations. Second, we could not differentiate whether the activation of predominantly alternative or lectin pathway is related to renal outcome and did not have the capacity to evaluate split products resulting from complement activation. Third, the immunosuppressive therapy has an impact on renal outcome and our study cohort is characterized by a heterogeneity in terms of treatment interventions. Thus, we could not fully account for this aspect in multivariate analysis. However, our study cohort was well characterized and had an adequate follow-up period that made possible the comparison of different histologic approaches in IgAN.

## Conclusions

In conclusion, there is a need to refine the histological assessment of IgAN, taking into account variables not included in the Oxford Classification, that could possibly suggest a different treatment approach. Accordingly, we have shown that the LM pattern of glomerular injury and the intensity of mesangial C3 deposition might stratify more accurately the renal outcome in patients with IgAN.

### Electronic supplementary material

Below is the link to the electronic supplementary material.

Supplementary Material 1

## Data Availability

All data generated or analyzed during this study are included in this published article [and its supplementary information files].

## Abbreviations

AAV: ANCA-associated vasculitis
C: crescents
CI: confidence interval
CKD: chronic kidney disease
E: endocapillary hypercellularity
eGFR: estimated glomerular filtration rate
ESRD: end-stage renal disease
GN: glomerulonephritis
HR: hazard ratio
IF: immunofluorescence
IFTA: interstitial fibrosis and tubular atrophy
IgAN: IgA nephropathy
IQR: interquartile range
IS: immunosuppressive
LM: light microscopy
LN: lupus nephritis
M: mesangial hypercellularity
MAP: mean arterial pressure
S: segmental sclerosis
SGLT2: sodium-glucose cotransporter 2
RAAS: renin-angiotensin-aldosterone system
T: tubular atrophy and interstitial fibrosis
TRF: targeted-release formulation
y: years
