# Supplementary material for: Histological reappraisal of IgA nephropathy: the role of glomerular pattern of injury and mesangial complement deposition
Source: BMC Nephrol. 2024 Apr 24;25:145. doi: 10.1186/s12882-024-03577-z (PMC11040743; doi:10.1186/s12882-024-03577-z)
Supplement: Supplementary file 1 — Supplementary Material 1 [file 12882_2024_3577_MOESM1_ESM.docx]

**Supplemental Table 1.** Univariate analysis regarding variables associated with the intensity of C3 staining or the composite endpoint.

| **Variable** | **No intense C3 staining** | **Intense C3 staining** | **p-value** | **No composite endpoint** | **Composite endpoint** | **p-value** |
| --- | --- | --- | --- | --- | --- | --- |
| ***Clinical parameters*** | | | | | | |
| **Age (years)** | 40.3 ± 13.1 | 40.8 ± 11.6 | 0.79 | 41.1 ± 13.6 | 42.1 ± 9.7 | 0.57 |
| **Sex (male, %)** | 61.4% | 69.1% | 0.29 | 64.5% | 71% | 0.36 |
| **Mean arterial pressure (mmHg)** | 96 ± 16 | 101 ± 13 | 0.09 | 97 ± 14 | 105 ± 14 | <0.001 |
| **Serum creatinine (mg/dL)** | 1.94 ± 1.6 | 2.08 ± 1.7 | 0.23 | 1.6 ± 1.13 | 3.06 ± 1.97 | <0.001 |
| **eGFR (ml/min/1.73m^2^)** | 60 ± 33 | 53 ± 31 | 0.16 | 65.4 ± 30.5 | 30.1 ± 15.8 | <0.001 |
| **CKD stage (%)** |  |  |  |  |  |  |
| - G1 | 22.9% | 13.6% | 0.36 | 22.4% | 0% | <0.001 |
| - G2 | 22.9% | 26.4% |  | 32.9% | 4.8% |  |
| - G3 | 32.9% | 31.8% |  | 31.6% | 40.3% |  |
| - G4 | 18.6% | 20% |  | 11.8% | 37.1% |  |
| - G5 | 2.9% | 8.2% |  | 1.3% | 17.7% |  |
| **Albumin (g/dL)** | 4.1 ± 0.6 | 4 ± 0.6 | 0.19 | 4.1 ± 0.6 | 3.8 ± 0.5 | <0.001 |
| **Serum IgA (mg/dL)** | 356 ± 130 | 348 ± 154 | 0.38 | 360 ± 160 | 322 ± 125 | 0.21 |
| **Increased serum IgA (% pf pts.)** | 31.9% | 23.4% | 0.32 | 31.3% | 16.7% | 0.11 |
| **Serum C3 (mg/dL)** | 116 ± 28 | 115 ± 22 | 0.8 | 118 ± 25 | 110 ± 22 | 0.04 |
| **Decreased serum C3 (% of pts.)** | 16.1% | 11.1% | 0.44 | 10.2% | 20% | 0.09 |
| **IgA/C3 ratio** | 3.1 ± 1.3 | 3.1 ± 1.4 | 0.72 | 3.2 ± 1.7 | 3.03 ± 1.1 | 0.88 |
| **Hematuria (cells/µL)** | 34 (11-74) | 36 (19-97) | 0.21 | 35 (13-91) | 30 (15-72) | 0.44 |
| **Hematuria (% of pts)** |  |  |  |  |  |  |
| - Absent | 37.7% | 33% | 0.77 | 33.8% | 45.8% | 0.32 |
| - Mild | 30.4% | 27.4% |  | 30.4% | 22% |  |
| - Moderate | 13% | 17% |  | 14.9% | 16.9% |  |
| - Severe | 18.9% | 22.6% |  | 20.9% | 15.3% |  |
| **24-h proteinuria (g/24h)** | 1.2 (0.5-3.1) | 1.7 (0.8-3.6) | 0.05 | 1.3 (0.6-2.8) | 2.6 (1.3-4.9) | <0.001 |
| **24-h proteinuria (% of pts)** |  |  |  |  |  |  |
| - Proteinuria < 0.75g/24 h(%) | 33.3% | 17% | 0.04 | 28.7% | 8.6% | 0.001 |
| - Proteinuria 0.75-3.5 g/24 h (%) | 44.4% | 57.5% |  | 53.8% | 55.2% |  |
| - Proteinuria >3.5 g/24 h (%) | 22.2% | 25.5% |  | 17.5% | 36.2% |  |
| ***Treatment*** | | | | | | |
| - RAAS blockade(%) | 85.1% | 89.7% | 0.47 | 88.7% | 86.2% | 0.62 |
| - Corticosteroids monotherapy (%) | 38.8% | 50.5% | 0.02 | 45.3% | 48.3% | <0.001 |
| - Corticosteroids ± other IS agents | 22.4% | 29.9% |  | 19.3% | 41.4% |  |
| ***Histologic parameters*** | | | | | | |
| **Global glomerulosclerosis (% pf glomeruli)** | 16.6% (0-33) | 20.7% (0-40.8) | 0.11 | 12.5% (0-28.9) | 33.3% (18.3-55) | <0.001 |
| **Oxford Classification (% of patients)** | | | | | | |
| **Mesangial hypercellularity (M1)** | 64.3% | 81.8% | 0.008 | 71.1% | 83.9% | 0.05 |
| **Endocapillary hypercellularity (E1)** | 25.7% | 23.6% | 0.75 | 22.4% | 29% | 0.3 |
| **Segmental sclerosis (S1)** | 51.4% | 64.5% | 0.08 | 55.9% | 64.5% | 0.24 |
| **IFTA (T)** |  |  |  |  |  |  |
| - T0 (≤25%) | 71.4% | 49.1% | 0.006 | 67.1% | 30.6% | <0.001 |
| - T1 (26-50%) | 21.4% | 30% |  | 25% | 33.9% |  |
| - T2 (>50%) | 7.1% | 20.9% |  | 7.9% | 35.5% |  |
| **Crescents (C)** |  |  |  |  |  |  |
| - C0 | 78.6% | 76.4% | 0.86 | 87.5% | 61.3% | <0.001 |
| - C1 | 14.3% | 17.3% |  | 9.9% | 24.2% |  |
| - C2 | 7.1% | 6.4% |  | 2.6% | 14.5% |  |
| **Intense C3 staining on IF** | - | - | - | 56.3% | 72.2% | 0.04 |
| **IgA staining on IF** |  |  |  |  |  |  |
| - Alone | 31.3% | 15.9% | 0.03 | 24% | 16.3% | 0.06 |
| - IgA+IgG co-deposition | 38.8% | 39.3% |  | 42.4% | 30.6% |  |
| - IgA+IgM co-deposition | 29.9% | 44.9% |  | 33.6% | 53.1% |  |
| ***Outcome*** | | | | | | |
| - Doubling of serum creatinine (%) | 8.6% | 23.6% | 0.01 | - | - | - |
| - ESRD (%) | 15.7% | 30.9% | 0.02 | - | - | - |
| - Combined endpoint (%) | 21.4% | 35.5% | 0.04 | - | - | - |
| ***eGFR decline**** |  |  |  |  |  |  |
| - eGFR change/y (ml/min/1.73m^2^/y) | +3.03 (-1.43 to 7.5) | -1.4 (-3 to 0.19) | 0.02 | - | - | - |
| - Percentage eGFR change (%) | +7.61% (-3.1 to 18.2) | -8.4% (-18.6 to 1.7) | 0.01 | - | - | - |
| - eGFR decline >5 ml/min/y (% of pts.) | 12.9% | 20.4% | 0.22 | - | - | - |

******* *Analysis after exclusion of patients with rapid progression to ESRD (≤12 months).*

***Abbreviations:*** *eGFR, estimated glomerular filtration rate; CKD, chronic kidney disease; RAAS, renin-angiotensin-aldosterone system; ESRD, end-stage renal disease; LM, light microscopy, M, mesangial hypercellularity; E, endocapillary hypercellularity; S, segmental sclerosis; IFTA, tubular atrophy and interstitial fibrosis; C, crescents; IF, immunofluorescence; MAP, mean arterial pressure; y, years; pts, patients; IS, immunosuppressive.*

**Supplemental Table 2.** Renal survival in relation to the intensity of mesangial C3 deposition and various histological variables.

|  |  | **1-y renal survival** | | | **5-year renal survival** | | |
| --- | --- | --- | --- | --- | --- | --- | --- |
|  | **Variable** | **Overall** | **No intense C3 staining** | **Intense C3 staining** | **Overall** | **No intense C3 staining** | **Intense C3 staining** |
| **M** | **M0** | 87.9% | 95% | 73.5% | 76.7% | 75.6% | 65.3% |
|  | **M1** | 91% | 90.4% | 90% | 74.4% | 78.4% | 70.7% |
| **E** | **E0** | 88.3% | 95.6% | 84.2% | 76.5% | 80.4% | 72.6% |
|  | **E1** | 94.1% | 81% | 92.3% | 71.2% | 72% | 61.9% |
| **S** | **S0** | 91.9% | 87.5% | 89.7% | 79.3% | 81% | 73.2% |
|  | **S1** | 89% | 96.6% | 85.7% | 71.8% | 74.9% | 67.5% |
| **T** | **T0** | 98.3% | 95.6% | 100% | 90.5% | 84.4% | 93% |
|  | **T1** | 87.5% | 92.3% | 87.9% | 64% | 55.4% | 62.1% |
|  | **T2** | 67.6% | 80% | 56.5% | 40.3% | 80% | 23.6% |
| **C** | **C0** | 92% | 100% | 86.8% | 81.6% | 87.7% | 75.4% |
|  | **C1** | 86.2% | 77.8% | 83.6% | 55.1% | 62.2% | 47.5% |
|  | **C2** | 76.9% | 60% | 100% | 36.9% | 20% | 53.6% |
| **IgA staining** | **IgA alone** | 91.1% | 100% | 82.4% | 81.2% | 77.8% | 82.4% |
|  | **IgA+IgG** | 98.5% | 100% | 97.6% | 84.6% | 82.9% | 85.5% |
|  | **IgA+IgM** | 82.3% | 89.7% | 79.2% | 63.7% | 79.2% | 58.4% |
| **LM pattern** | **Normal glomeruli** | 100% | 100% | 100% | 100% | 100% | 100% |
|  | **Mesangioproliferative** | 94.8% | 95.5% | 93.3% | 90.7% | 90.7% | 88.6% |
|  | **Proliferative/necrotizing** | 91.3% | 91.7% | 95.8% | 71.2% | 62.5% | 71.6% |
|  | **Crescentic** | 85.7% | 66.7% | 75% | 42.9% | 33.3% | 50% |
|  | **Sclerosing** | 74.7% | 100% | 63% | 48.8% | 83.3% | 34.5% |

***Abbreviations:*** *LM, light microscopy, M, mesangial hypercellularity; E, endocapillary hypercellularity; S, segmental sclerosis; T, tubular atrophy and interstitial fibrosis; C, crescents.*
